# Anti-proliferative potential of Glucosamine in renal cancer cells via inducing cell cycle arrest at G0/G1 phase

**DOI:** 10.1186/s12894-017-0221-7

**Published:** 2017-05-30

**Authors:** Long-sheng Wang, Shao-jun Chen, Jun-feng Zhang, Meng-nan Liu, Jun-hua Zheng, Xu-dong Yao

**Affiliations:** Department of Urology, Shanghai Tenth People’s Hospital, Tongji University, School of Medicine, Shanghai, 200072 China

**Keywords:** Renal cell carcinoma, Glucosamine, Proliferation, Cell cycle

## Abstract

**Background:**

Renal cell carcinoma (RCC) is one of the most common types of cancer in urological system worldwide. Recently, the anticancer role of Glucosamine has been studied in many types of cancer. The aim of this study was to investigate the effects of Glucosamine on RCC.

**Methods:**

The effects of Glucosamine on RCC cell proliferation and apoptosis were investigated by MTT assay and Annexin V-FITC Apoptosis assay, respectively in vitro. Cell cycle was detected by flow cytometry after treatment with Glucosamine. Protein levels of several cell cycle associated markers were examined by Western Blot.

**Results:**

Our data showed that Glucosamine significantly inhibited the proliferation of renal cancer 786-O and Caki-1 cells in a dose-dependent manner. Besides, Glucosamine treatment resulted in cell cycle arrest at G0/G1 phase in both cell lines. Meanwhile, the expression of several regulators that contribute to G1/S phased transition, such as Cyclin D1, CDK4 and CDK6, were significantly down-regulated with the up-regulation of cell cycle inhibitors, p21 and p53, after treatment with glucosamine. However, the apoptosis rate of RCC cells was down-regulated when treatment with Glucosamine at 1 mM and 5 mM, while up-regulated at 10 mM.

**Conclusions:**

Our findings indicated that Glucosamine inhibited the proliferation of RCC cells by promoting cell cycle arrest at G0/G1 phase, but not promoting apoptosis. The present results suggested that Glucosamine might be a potential therapeutic agent in RCC treatment in the future.

## Background

D-Glucosamine (2-amino-2-deoxy-d-glucose), a naturally occurring amino monosaccharide, is used to synthesize UDP-GlcNAc in the body via the hexosamine biosynthetic pathway (HBP) [1]. UDP-GlcNAc is the principal substrate for the glycosylation of proteins [2, 3]. Because it is highly water soluble and nontoxic, Glucosamine has been widely used as a nutritional supplement in both humans and dogs [4]. The most famous use of glucosamine is to treat human osteoarthritis [5, 6]. Except for its chondroprotective action, glucosamine has been demonstrated to have many other functions, such as anti-cancer.

Glucosamine was first demonstrated as an anticancer pharmaceutical more than 60 years ago [7]. Recently, more attentions have been attracted to its antitumor properties. Glucosamine is an effective lytic agent for several types of tumors with little toxicity to normal cells [8]. It has been reported that glucosamine suppresses the proliferation of human prostate cancer DU145 cells through STAT3 signaling pathway [9]. Glucosamine can also play anti-cancer activity through the inhibition of N-linked glycosylation [10]. In retinal pigment epithelial cells, glucosamine exhibits its antitumor role through the inhibition of epidermal growth factor-induced proliferation and cell-cycle progression [11]. However, the effects of Glucosamine on renal cancer cells remain unclear, let alone the mechanisms.

It is well-known that cell cycle deregulation is one of the most prevalent characteristics of all cancers [12]. Cyclins, cyclin-dependent kinases (CDKs) and various kinds of cyclin-dependent kinase inhibitors (CDKIs) are closely involved in cell cycle distribution [13]. Cyclin D1, which is responsible for the transition from G1 phase to S phase via forming complexes with CDK4 and CDK6, is one of the most important cell cycle regulators in the process of tumor development [14, 15]. M. S. Lima et al. reported that Cyclin D1 was overexpressed in RCC and can be used as an prognostic factor in RCC patients [16]. P21 protein is a well-known CDK inhibitor, participating in cell-cycle regulation via the inhibition of cyclin-CDK complex activity in G1 phase [15]. Tumor suppressor p53 also takes part in regulating the cell cycle process and p21 acts as a major downstream effector of p53 in G1 phase [17, 18]. The deficiency of p21 along with p53 may lead to unrestricted cell cycle progression and carcinogenesis.

Renal cell carcinoma, which accounts for about 3% of all cancers in adults, is one of the most common diagnosed cancers in urological system in the world [19]. About 62,700 cases of kidney cancer and renal pelvis cancer are expected to occur and lead to more than 14,240 deaths in the United States in 2016 [20]. Among all the subtypes of RCC, about 70% is clear cell histopathology [21]. It is estimated that approximate 25% of patients with RCC have encountered metastases at the time of diagnosis and another 25% have locally advanced disease [22]. RCC was reported inherently resistant to cytotoxic therapy, hormone therapy or radiation [23, 24].

The aim of the present study was to investigate the effects of D-Glucosamine on RCC cells. Our results show that Glucosamine inhibited the proliferation of renal cancer cells in a dose-dependent manner. Meanwhile, we tried to explore how Glucosamine exert its anti-proliferation effect. The apoptosis assay revealed that RCC cells apoptosis was down-regulated when treatment with Glucosamine at low concentration, while up-regulated at very high concentration. Moreover, Glucosamine treatment resulted in cell-cycle arrest in G0/G1 phase. Mechanically, Cyclin D1 was dramatically repressed by Glucosamine, while the expression of p53 and p21 was significantly up-regulated. These results suggested that Glucosamine inhibited the proliferation of renal cancer cells by promoting cell-cycle arrest.

## Methods

### Cell culture and reagents

Human renal cancer cell lines 786-O and caki-1 were purchased from the Cell Bank of Type Culture Collection of Chinese Academy of Sciences (CCCAS, China) and were cultured in RPMI-1640 medium (HyClone) supplemented with 10% fetal bovine serum (Gibco). All cells were cultured in a humidified incubator with 5% CO_2_ at 37 °C. D-glucosamine was purchased from Sigma Chemical Co (sigma A3286). All the antibodies were purchased from Abcam.

### MTT assay

Five groups of RCC cells were plated in 96-well culture plates in triplicate at a concentration of about 2 × 10^3^/well. 200 μl culture medium containing various concentrations of glucosamine (0 mM, 1 mM, 5 mM and 10 mM) was added into 96-well culture plates, respectively. After incubated for different time perious (12, 24, 48 and 72 h), 200 μl MTT solution was added to each well. Then, cells were incubated at 37 °C for 4 h and the medium was discarded from each well. After that, 200 μl DMSO was used and thoroughly mixed for 15 min. The optical density (OD) of each well was measured at 490 nm using a micro-plate reader (Bio-Rad). All experiments were performed three times and three replicates in each repeat.

### Western blot assay

Total protein was extracted using precooled RIPA lysis buffer with protease inhibitors. The concentration of total protein was measured using a Bio-Rad protein assay system. Equal amount of protein was separated by 10% SDS-PAGE for electrophoresis and transferred to nitrocellulose membranes (Bio-Rad). Afterward, the membrane was incubated at 4 °C for 12 h with specific primary antibodies (Bioworld, Nanjing, China). After incubation with secondary antibodies for 1 h at room temperature, signals were visualized.

### Cell cycle assays

To measure cell cycle distribution, the cells were harvested after addition with different concentrations of glucosamine for 24 h and fixed in 70% ice-cold ethanol overnight. Then, the cells were washed by PBS and stained with propidium iodide (PI; BD Biosciences) in PBS added with RNase (100 μg/ml) and Triton X-100 (0.2%) for 30 min. Afterward, cell cycle distribution was analyzed by flow cytometry according to the manufacturer’s guidelines (FACS, BD Biosciences). Tests were performed three times for each sample.

### Apoptosis assay

For the apoptosis assay, the Annexin V-FITC Apoptosis Detection kit (BD Biosciences) was used according to manufacturer’s protocol. Cells were serum starved for 24 h, and treated with various doses of glucosamine (0 mM, 1 mM, 5 mM and10 mM) for 24 h. RCC cells were collected and washed twice with PBS. Then, cells were re-suspended, stained with (FITC)-Annexin V/PI for 15 min and analyzed by flow cytometry (FACS, BD Biosciences).

### Statistical analysis

SPSS version 18.0 software was used for all statistical analyses of this study. Data are expressed as mean ± SD from at least three independent experiments. The differences between each experimental group was analyzed by Student’s *t*-test or chi-square test. *P*-value of <0.05 was treated as statistically significant.

## Results

### Glucosamine inhibits renal cancer cell proliferation

**Fig. 1:**
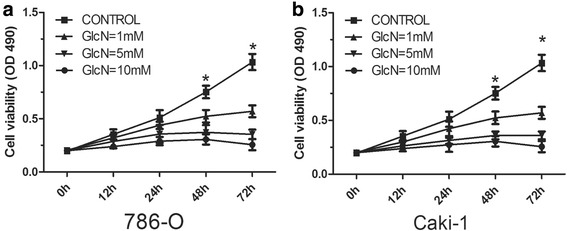
Glucosamine induces growth inhibition in 786-O (**a**) and Caki-1 cells (**b**). Cells were treated with various concentrations of Glucosamine (0 mM, 1 mM, 5 mM and 10 mM), and cell viability was analyzed using MTT assay at different time points (0 h,12 h, 24 h, 48 h and 72 h). * *P* <0.05 compared with the control group (0 mM)

To examine the effects of Glucosamine on the proliferation of human renal cancer cells, 786-O and Caki-1 cells were treated with Glucosamine at different doses and MTT assay was performed. Our data showed that Glucosamine inhibited the proliferation of RCC cells in a dose-dependent manner (Fig. 1). These results suggested that Glucosamine played an important anti-proliferative role in RCC cells.

### Effects of Glucosamine on cell apoptosis

**Fig. 2:**
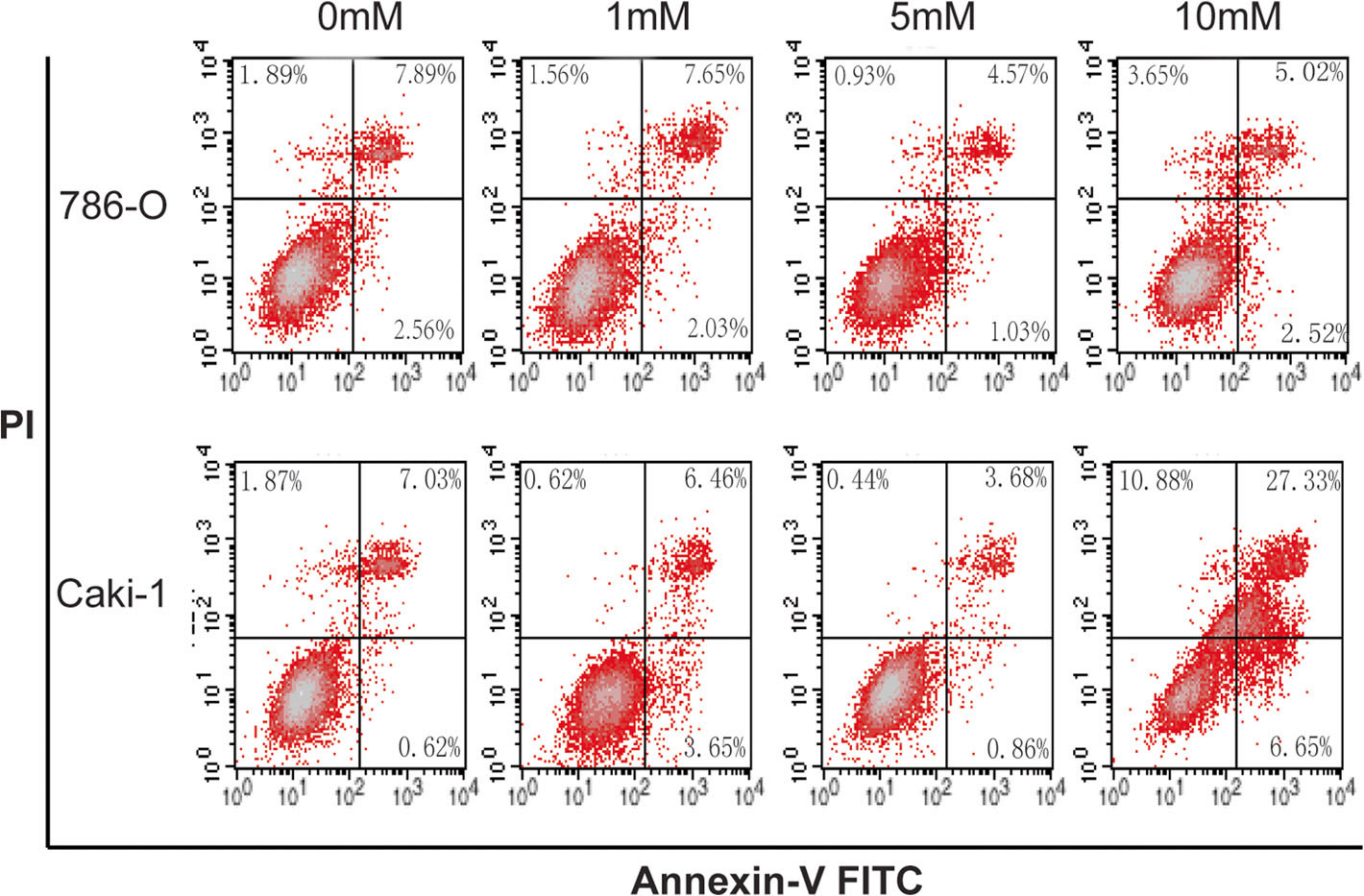
Effects of Glucosamine on the apoptosis of 786-O and Caki-1 cells as shown by Annexin V-FITC/PI analysis. 786-O and Caki-1 cells were treated for 24 h with various doses of Glucosamine under serum-free conditions, and apoptotic cells were measured as the percentage of Annexin V-positive*/*PI*-*negative cells. The representative images were shown. Three independent experiments were performed and the trend is the same

Previous studies have reported that Glucosamine induced apoptosis in various cell lines [25, 26]. Therefore, we investigated whether Glucosamine exerted anti-cancer role via inducing apoptosis in renal cancer cells lines. As shown in Fig. 2, the apoptosis rate of both cell lines was up-regulated by high concentration of Glucosamine (10 mM), but down-regulated by low concentrations of Glucosamine (1 mM and 5 mM), as compared with control group. These data suggested that low doses of Glucosamine-mediated proliferation inhibition of renal cancer cells was not due to apoptosis.

**Fig. 3:**
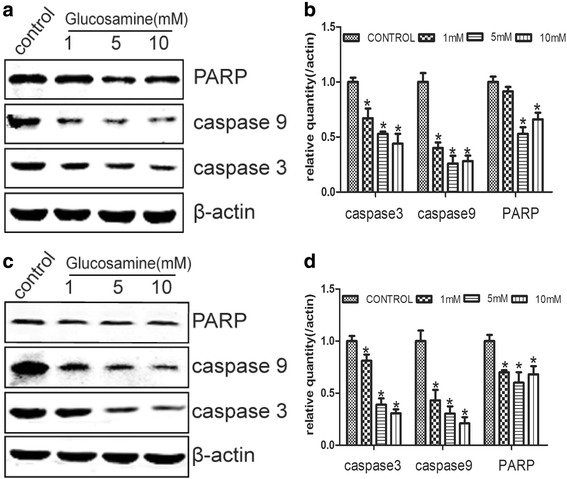
Effects of Glucosamine on the expression of apoptosis regulators caspase 3/9 and PARP. 786-O and Caki-1 cells were deprived of serum for 24 h and cultured with various doses of Glucosamine for 24 h. Afterward, the total protein was collected and the expression of caspase 3/9 and PARP was detected with Western Blot. The expression of these three proteins was obviously down-regulated in both 786-O (**a**) and Caki-1(**c**) cells. Columns show the mean values of three experiments of 786-O (**b**) and Caki-1(**d**) (± SD). *P < 0.05 compared with the control group (0 mM)

Members of caspases play vital role in the apoptotic process. The nuclear DNA repair enzyme poly (ADP-ribose) polymerase (PARP) is a target of caspase-3 and its cleavage is a biomarker for cell apoptosis [27, 28]. Thereby, we detected the expression of caspase-3, caspase-9 and PARP in RCC cells by Western blot. Our results showed that the protein levels of caspase-3, caspase-9 and PARP were significantly down-regulated by Glucosamine as compared with the control in both 786-O and Caki-1 cells (Fig. 3). These results were in line with the results of Annexin V-FITC Apoptosis assay. All these results indicated that Glucosamine inhibited the proliferation of RCC cells was not by inducing apoptosis.

### Glucosamine induces cell cycle arrest in RCC cells in a dose-dependent manner

**Fig. 4:**
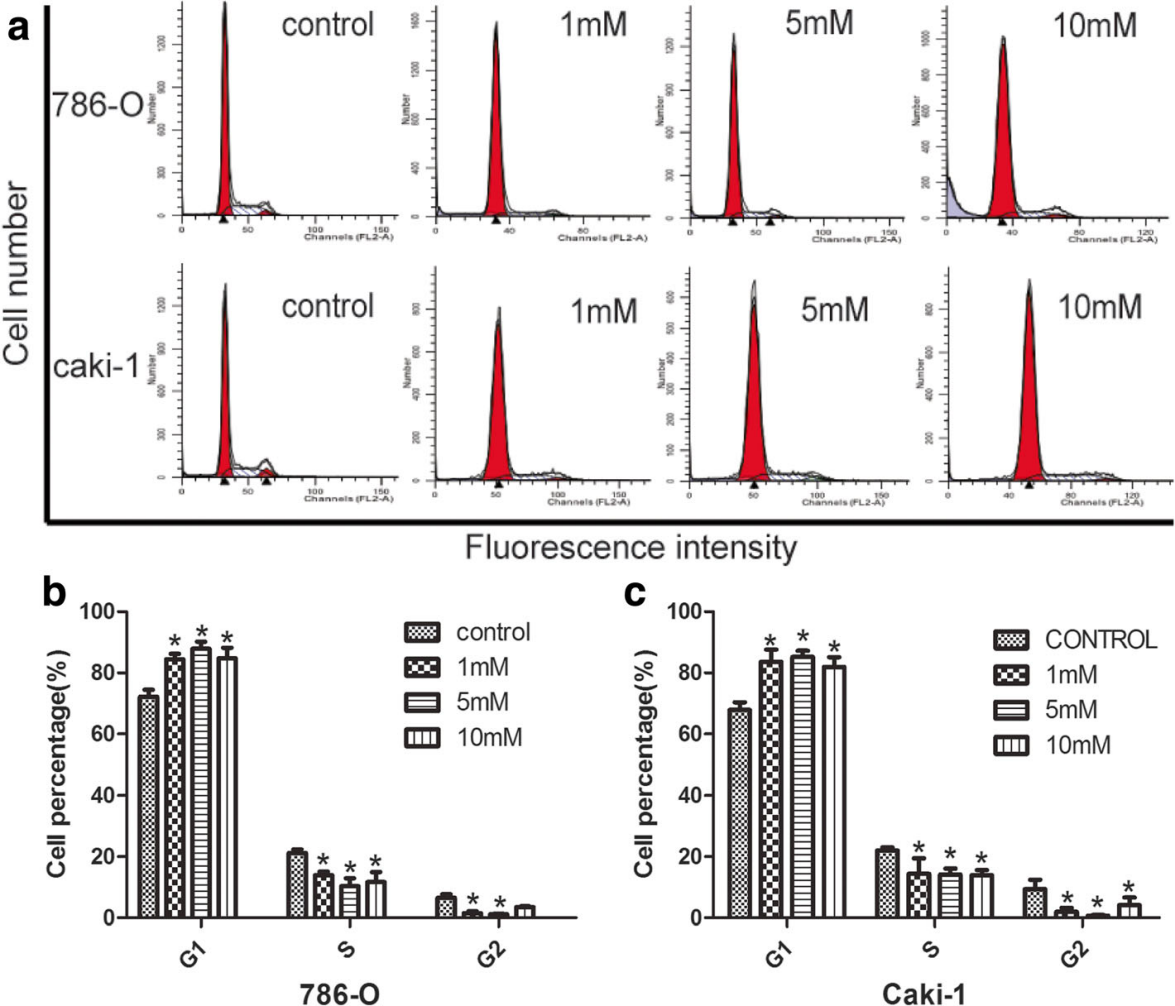
Effects of Glucosamine on cell-cycle progression in human renal cancer cell lines (786-O and Caki-1). **a** Cell cycle distribution of 786-O and Caki-1 cells was examined after treatment with various concentrations of Glucosamine for 24 h. **b**, **c** Columns show the mean values of three experiments (± SD). *P < 0.05 compared with the control group (0 mM)

Glucosamine could cause cell-cycle arrest in various types of cancer cell lines [29, 30]. To determine whether the anti-proliferation effect of Glucosamine was accompanied by the alteration in cell cycle process, we next investigated the cycle distribution of 786-O and caki-1 cells after Glucosamine treatment (0 mM, 1 mM, 5 mM, and 10 mM) for 24 h. As shown in Fig. 4, with the increasing doses of Glucosamine, G0/G1 cell population was gradually increased with the decrease of cells in S and G2/M phases. These results indicated that Glucosamine-mediated cell growth inhibition occurred at the G0/G1 to S transition phase.

### Down-regulation of Cyclin D1 and CDK4/6 by glucosamine in RCC cells

**Fig. 5:**
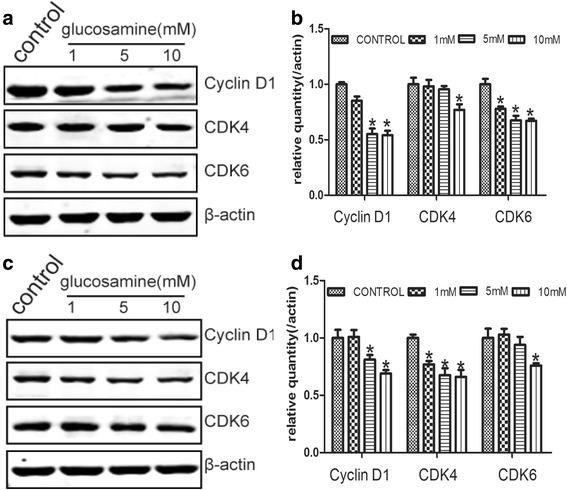
The expression of Cyclin D1, CDK4 and CDK6 were down-regulated by Glucosamine. When 786-O (**a**, **b**) and Caki-1 (**c**, **d**) cells were treated with Glucosamine for 24 h, the expression of Cyclin D1, CDK4 and CDK6 were down-regulated. Cells were lysed with RIPA lysis buffer, and the lysates were then analyzed by Western blot. **a**, **c** Representative gels from 3 experiments are shown. **b**, **d** Densitometric analysis was performed for each protein band relative to β-actin in the same sample using Quantity one software. *P < 0.05 compared with the control group (0 mM)

Cyclin D1 was reported to be a key element in cell proliferation in many types of cancers [31]. Meanwhile, on account of CDK4 and CDK6 preferably associate with the D type cyclins during the G1 phase [32], the expression of Cyclin D1, CDK4 and CDK6 were examined. Western blot results demonstrated that Cyclin D1 expression significantly repressed by Glucosamine with the dose raising (Fig. 5). Simultaneously, CDK4 and CDK6 were also gradually suppressed by Glucosamine in a dose-dependent manner (Fig. 5).

### Up-regulation of p53 and p21 by glucosamine in RCC cells

**Fig. 6:**
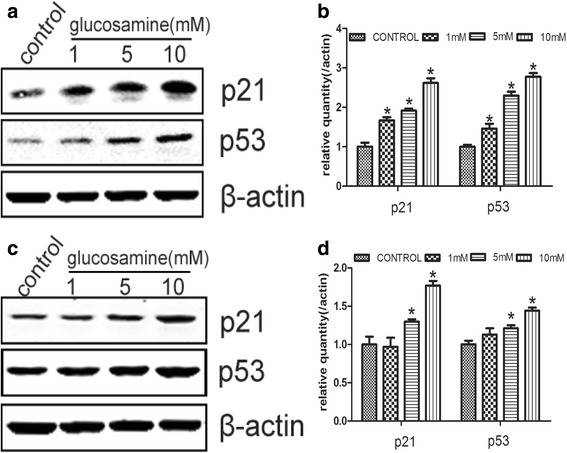
Glucosamine affects the expression of p21 and p53 in 786-O and Caki-1 cells. The expression of p21 and p53 was tested by Western blot after 786-O and Caki-1 cells were treated with various concentrations of Glucosamine for 24 h. β-actin served as internal loading control. **a**, **c** Representative gels from 3 experiments are shown. **b**, **d** Densitometric analysis was performed for each protein band relative to β-actin in the same sample using Quantity one software. *P < 0.05 compared with the control group (0 mM)

It is well known that the complexes of cyclins and CDKs, which is essential to the transition from G0/G1 to S phase, are inhibited by CDKIs [33]. p21 is one of the most important CDKI for CDK4/6. p53, a well-known tumor suppressor, can regulate cell cycle process by affecting the expression of p21 [17]. Thus, we examined the expression of p21 and p53 under the effect of Glucosamine. Glucosamine was found to gradually up-regulate the expression of p21 and p53 in protein levels (Fig. 6).

## Discussion

Renal cell carcinoma is one of the deadliest urogenital malignancies. The morbidity of RCC is increasing annually and the causes are multifactorial [34]. Abnormal cell cycle progression, such as shortening of the G1-phase, is involved in tumorigenesis.

Glucosamine and its derivatives are obligatory structural components of many biologically important macromolecules, such as membrane glycoproteins and mucopolysaccharide [35]. Although the anticancer property of glucosamine has been reported more than 60 years ago [7], the molecular mechanism remained unclear. Recently, Glucosamine has been demonstrated to be an effective anticancer agent in prostate cancer, lung cancer, leukemia and colorectal cancer [2, 25, 36, 37]. However, its action in renal cell carcinoma has not been studied.

In the present study, we analyzed the anticancer role of Glucosamine in renal cancer cells. We demonstrated that Glucosamine functioned as an anti-proliferation drug in renal cancer 786-O and Caki-1 cells. Afterward, we tried to certify whether the apoptosis and cell cycle of RCC cells were influenced. Using Annexin V-FITC Apoptosis assay, we found the apoptosis of RCC cells was down-regulated significantly when treated with low dose of Glucosamine and was up-regulated only in a very high dose. Likewise, several apoptosis markers were down-regulated obviously, such as caspase-9, caspase-3 and PARP. Notably, Glucosamine induced G0/G1 arrest in the process of RCC cell cycle. Meanwhile, results of Western blot revealed significant down-regulation of Cyclin D1, CDK4, and CDK6 and up-regulation of p21 and p53 under treatment of Glucosamine, especially at the dose of 10 mM comparing with untreated samples. These results indicated that Glucosamine could exert its anticancer affect in RCC cells via causing cell cycle arrest and up-regulating cancer suppressor gene p21 and p53. However, the potential detailed mechanisms involved in the anti-tumor property of Glucosamine in RCC are required to be revealed in the future.

Our results implied that the antiproliferation role of Glucosamine in RCC cells was not related to apoptosis, and this result was consistent with the findings of Chang-Min Liang et al. [11]. They reported that Glucosamine did not induce apoptosis in the ARPE-19 cells, but inhibit epidermal growth factor-induced proliferation by causing cell cycle arrest. However, Zhe Wang et al. reported that Glucosamine sulfate induced apoptosis in chronic myelogenous leukemia K562 cells and this regulation was associated with translocation of cathepsin D and downregulation of Bcl-xL [25]. Otherwise, Ki-Hoon Song et al. showed that Glucosamine induced cell cycle arrest and apoptosis in NSCLC cells [26]. We assume that Glucosamine may play various roles in different types of cancers.

## Conclusion

Current study found that, Glucosamine inhibited the proliferation of RCC cells mostly by causing cell cycle arrest at G0/G1 phase. Glucosamine treatment in RCC cells led to the down-regulation of Cyclin D1, CDK4 and CDK6, as well as the up-regulation of p21 and p53. Cumulatively, these findings indicate that Glucosamine might serve as a potential therapeutic in the future.

## Abbreviations

CDK: Cyclin-dependent kinases
HBP: Hexosamine biosynthetic pathway
PARP: Poly (ADP-ribose) polymerase
RCC: Renal cell carcinoma
